# Fertility preservation in male adolescents with cancer (2011–2020): A retrospective study in China

**DOI:** 10.1002/cam4.7354

**Published:** 2024-06-13

**Authors:** Shasha Liu, Qiling Wang, Wenbing Zhu, Zhou Zhang, Wenhao Tang, Huiqiang Sheng, Jigao Yang, Yushan Li, Xiaowei Liang, Tianqing Meng, Zhiqiang Wang, Faxi Lin, Hao Dong, Xiaojin He, Xianglong Jiang, Shanjun Dai, Aiping Zhang, Chunying Song, Zuowen Liang, Feng Zhang, Xiaojun Wang, Peiyu Liang, Guihua Gong, Xiaohong Huai, Yanyun Wang, Fuping Li, Xinzong Zhang

**Affiliations:** ^1^ Human Sperm Bank, Key Laboratory of Birth Defects and Related Diseases of Women and Children of Ministry of Education, West China Second University Hospital Sichuan University Chengdu Sichuan China; ^2^ NHC Key Laboratory of Male Reproduction and Genetics, Guangdong Provincial Reproductive, Science Institute, Guangdong Provincial Fertility Hospital Guangzhou Guangdong China; ^3^ Reproductive and Genetic Hospital CITIC Xiangya Changsha Hunan China; ^4^ Northwest Women and Children's Hospital Xian Shaanxi China; ^5^ Department of Urology Peking University Third Hospital Beijing China; ^6^ Zhejiang Mater Child and Reproductive Health Center Zhejiang Hangzhou China; ^7^ Human Sperm Bank Chongqing Research Institute for Population and Family Planning Science and Technology Chongqing China; ^8^ Henan Human Sperm Bank The Third Affiliated Hospital of Zhengzhou University Zhengzhou Henan China; ^9^ Human Sperm Bank of National Research Institute for Family Planning Beijing China; ^10^ Hubei Province Human Sperm Bank, Center for Reproductive Medicine, Tongji Medical College Huazhong University of Science and Technology Wuhan Hubei China; ^11^ The First Affiliated Hospital of Guangxi Medical University Guilin Guangxi China; ^12^ The First Affiliated Hospital of Nanjing Medical University Nanjing Jiangsu China; ^13^ Department of Urological Surgery First Affiliated Hospital of Kunming Medical University Kunming Yunnan China; ^14^ Anhui Provincial Human Sperm Bank The First Affiliated Hospital of Anhui Medical University Hefei Anhui China; ^15^ Jiangxi University of Traditional Chinese Medicine Nanchang Jiangxi China; ^16^ The First Affiliated Hospital of Zhengzhou University Zhengzhou Henan China; ^17^ Human Sperm Bank of The First Hospital of Lanzhou University Lanzhou Gansu China; ^18^ Sperm Bank, Shanxi Bethune Hospital Shanxi Academy of Medical Sciences Taiyuan Shanxi China; ^19^ The First Hospital of Jilin University Jilin China; ^20^ Obstetrics and Gynecology Hospital Fudan University Shanghai China; ^21^ Maternal and Child Health Hospital of Urumqi Xinjiang China; ^22^ The First Affiliated Hospital of Hainan Medical University Haikou Hainan China; ^23^ Human Sperm Bank of Chifeng Gynecology and Obstetrics Hospital Chifeng Inner Mongolia China; ^24^ Liaoning Maternal and Child Health Hospital Shenyang Liaoning China; ^25^ Laboratory of Molecular Translational Medicine, Center for Translational Medicine, Key Laboratory of Birth Defects and Related Diseases of Women and Children (Sichuan University), West China Second University Hospital Sichuan University Chengdu Sichuan China

**Keywords:** cancer, cryopreservation, fertility preservation, male adolescents, semen quality

## Abstract

**Background:**

According to the studies, more than 80% of pediatric patients with cancer can achieve a survival rate greater than 5 years; however, long‐term chemotherapy and/or radiation therapy may seriously affect their reproductive ability. Fertility preservation in adolescents with cancer in China was initiated late, and related research is lacking. Analyze data to understand the current situation and implement measures to improve current practices.

**Methods:**

From 2011 to 2020, data on 275 male adolescents with cancer whose age ranged from 0 to 19 years old were collected from 16 human sperm banks for this retrospective study. Methods include comparing the basic situation of male adolescents with cancer, the distribution of cancer types, and semen quality to analyze the status of fertility preservation.

**Results:**

The mean age was 17.39 ± 1.46 years, with 13 cases (4.7%) aged 13–14 years and 262 cases (95.3%) aged 15–19 years. Basic diagnoses included leukemia (55 patients), lymphomas (76), germ cell and gonadal tumors (65), epithelial tumors (37), soft tissue sarcomas (14), osteosarcoma (7), brain tumors (5), and other cancers (16). There are differences in tumor types in different age stages and regions. The tumor type often affects semen quality, while age affects semen volume. Significant differences were found in sperm concentration and progressive motility before and after treatment (*p* < 0.001). Moreover, 90.5% of patients had sperm in their semen and sperm were frozen successfully in 244 patients (88.7%).

**Conclusions:**

The aim of this study is to raise awareness of fertility preservation in male adolescents with cancer, to advocate for fertility preservation prior to gonadotoxic therapy or other procedures that may impair future fertility, and to improve the fertility status of future patients.

## 1 INTRODUCTION

Cancer has been identified as one of the most common causes of death in young people. A Chinese study reported that between 1991 and 2010, the incidence of malignant tumors in male adolescents aged 5–19 in Dalian was 11.79/100,000.^1^ From 2006 to 2009 in China, the incidence of malignant tumors in male adolescents aged 0–14 years was 10.265/100,000, which was higher than that in females (9.18/100,000). The latest national cancer report by the China Cancer Center in 2018 found that in 2014, the number of new cancer cases in male patients aged 0–14 was 12,900. Between 2018 and 2020, newly diagnosed cancer cases among children and adolescents in China were approximately 40,000 cases per year, there were approximately 52,478 male patients aged 0–14 years and 15,690 patients aged 15–19 years.^2^ In recent years, the number of reported cancers in children and adolescents has been on the rise. In 2020, there will be approximately 20,248 new male cancer cases among children and adolescents aged 0–19 years in China, of which about 15,678 cases will be in the age group of 0–14 years and about 4570 cases in the age group of 15–19 years. The number of new cancers in males aged 0–14 years is about four times higher than in those aged 15–19 years (data from Cancer Today https://gco.iarc.fr/today/online‐analysis). In the 1960s, only a few adolescents and young adults survived their initial cancer diagnoses; thus, little emphasis was placed on fertility preservation.^3^ This dramatic improvement in survival is primarily due to enhanced multimodal treatments and combined chemotherapeutic regimens. The 5‐year survival of adolescents and young adults with all cancers has been estimated to be at 75%–90%.^4^, ^5^, ^6^, ^7^, ^8^, ^9^, ^10^

However, malignant diseases and their treatments (surgery, radiation, and/or chemotherapy) will either transiently or permanently affect future fertility.^11^ The prepubertal testis appears to be more sensitive to oncological treatments than the adult testis, as the testicular environment is not quiescent, but rather, there is constant turnover of early germ cells.^12^ Older studies report a rather high rate (66%–83%) of infertility in survivors of childhood cancer.^13^, ^14^, ^15^, ^16^, ^17^ The American Society of Clinical Oncology (ASCO), the National Comprehensive Cancer Network (NCCN), the Japan Society of Clinical Oncology (JSCO), the American Academy of Pediatrics (AAP), the European Society of Human Reproduction and Embryology (ESHRE), and the European Society for Medical Oncology (ESMO) promote the importance of fertility protection for male adolescents affected by cancer. The goal of male fertility protection is to preserve sperm before treatment, but fertility protection care is still stagnant. Studies have reported that lack of communication, the urgency to begin treatment, high cost, parental attitudes, the knowledge gap of the provider, and the doctor's recommendation can all affect whether fertility preservation is performed before treatment.^18^, ^19^, ^20^, ^21^, ^22^, ^23^, ^24^, ^25^ However, fertility preservation in children and adolescents with cancer in China was initiated late. In order to improve the quality of life in adolescent cancer survivors, we need to fully understand the current status of fertility preservation, analyze the problems faced in depth, and solve them. This study reviewed the fertility preservation status in China over the past 10 years, and with a combination of current research reports, the aim is to promote continuous clinical summary and accumulation experience, enhance and perfect fertility preservation in children and adolescents with cancer, and provide a guarantee for patients' fertility.

## 2 METHODS

### 2.1 Patient population

Of the 27 human sperm banks in China, 2 have not uploaded information, and only 16 were known to have performed fertility preservation in male adolescents with cancer. The 16 human sperm banks are located in three provinces in eastern region, seven provinces in central region, and six provinces in western region. The provinces are Beijing, Zhejiang, Guangdong, Jilin, Shanxi, Henan, Jiangxi, Anhui, Hubei, Hunan, Sichuan, Yunnan, Chongqing, Shaanxi, Gansu, and Guangxi.

From 2011 to 2020, data on 275 male adolescents with cancer whose age ranged from 0 to 19 years old were collected from 16 human sperm banks for this retrospective study. Men aged 0–19 years who were diagnosed with tumors and who attempted to go to a human sperm bank for fertility preservation before and after treatment were included in the study, regardless of whether they ultimately succeeded in cryopreserving their spermatozoa. We divided the patients into two groups: the 0–14 years of age and the 15–19 years of age groups. Cancer in adolescents and young adults (AYAs) is defined by the National Cancer Institute as diagnoses occurring among those aged 15–39 years.^26^ The 15–19 years of age is generally considered to be postpubertal.^3^ The 0–14 years of age is generally considered to be prepubertal and pubertal.

In accordance with the rules and procedures in code of practice formulated by the council on Human Reproductive Technology, the management of patients was similar in 16 human sperm banks. After presentation by a specialized staff, patients fully informed about the process of fertility preservation, costs, future use, and storage duration. It was stipulated that the stored gametes were only used for married couples. No posthumous conception would be allowed. After receiving written and oral consent, the patients were required to sign a cryostorage consent. The study was approved by the Ethic Committee of West China Second University Hospital, Sichuan University (No: 2020‐001) and was conducted in accordance with the Declaration of Helsinki. The type of cancer was determined from the histological diagnosis. Records included basic patient information (age, ethnicity, place of residence, tumor type, and fertility‐related topics), sperm parameters (abstinence time, semen volume, sperm concentration, and sperm progressive motility before and after freezing), and azoospermia rates.

Semen samples were collected through masturbation. Semen analysis was performed after liquefaction for 30 min at 37°C in accordance with WHO guidelines (World Health Organization, 2010).^27^ Azoospermia was confirmed by centrifugation of the entire semen sample at 3000 × g for 15 min. Sperm specimens were frozen by adding cryoprotectant containing glycerol and thawed at 37°C for use. If no motile sperm were detected before or after freezing, the sample would not be banked after notifying the patients. As only a few sperm are required for in vitro fertilization (IVF) with intracytoplasmic sperm injection (ICSI), any sample containing motile sperm was banked.

### 2.2 Statistical analysis

The achieved outcomes were analyzed using the Statistical Package for the Social Sciences version 19.0 (IBM, New York, USA). Results were expressed as the mean ± standard deviation, and *p* < 0.05 was considered statistically significant. Datasets were analyzed using chi‐squared test, independent sample *t*‐test, ANOVA, and *t*‐test.

## 3 RESULTS

### 3.1 Study population

**FIGURE 1:**
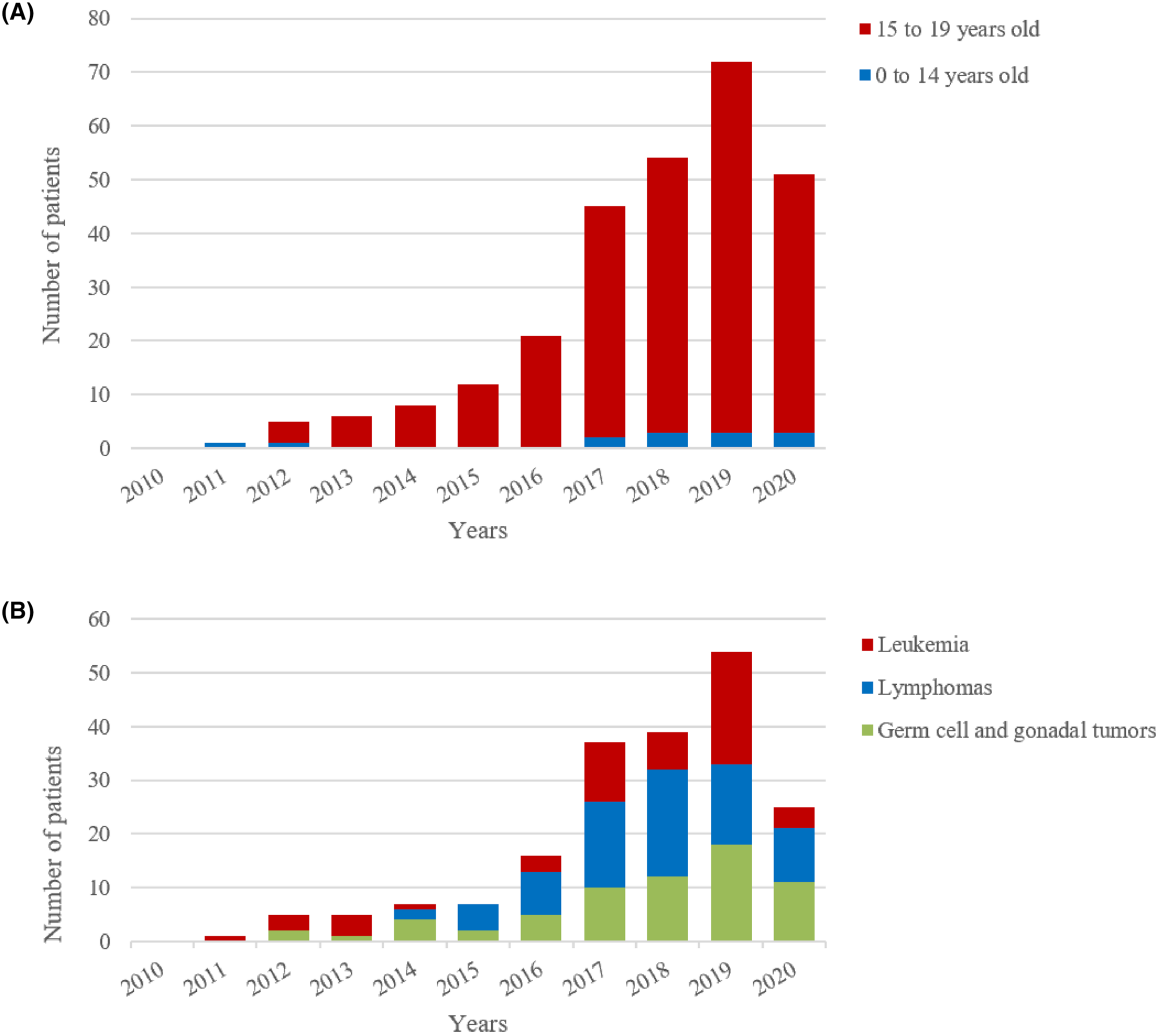
Number of patients with (A) the two age groups (13–14, 15–19 years old), (B) the three most common cancer types (leukemia, lymphoma, and germ cell and gonadal tumors) undergoing sperm cryopreservation from 2011 to 2020.

**FIGURE 2:**
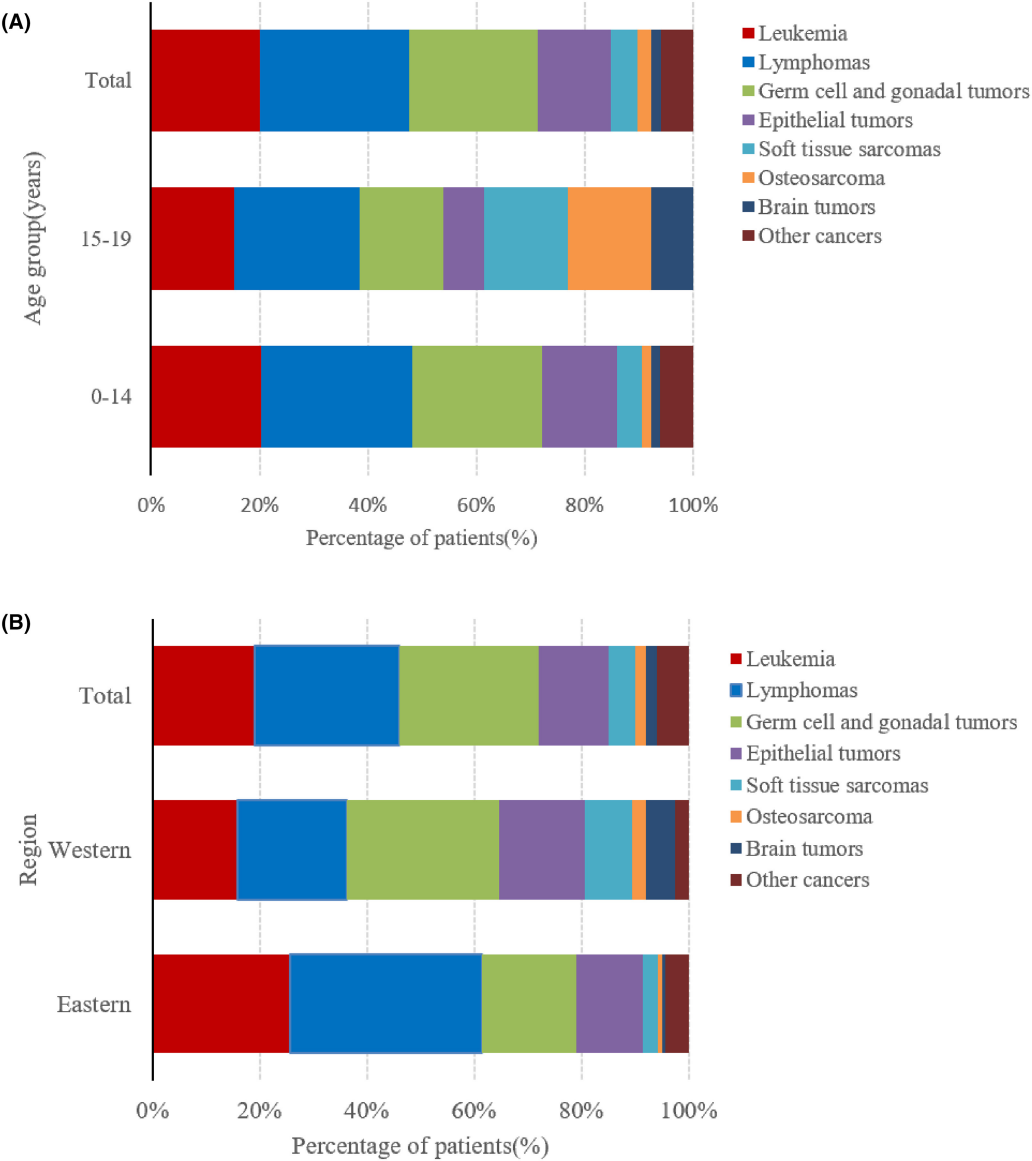
(A) Proportional distribution of cancer type by age group. (B) Proportion of patients with the listed cancer types undergoing sperm cryopreservation in eastern and western China.

During the study period, 275 male adolescent patients with cancer, whose age ranged from 0 to 19 years old (average 17.39 ± 1.46), visited sperm bank for fertility preservation. Thirteen patients (4.7%) were aged 0–14 years, and 262 patients (95.3%) were aged 15–19 years. The number of patients who have undergone fertility preservation has increased gradually with time and declined slightly in 2020 due to COVID‐19 (Figure 1A). Basic diagnoses included 55 cases of leukemia (19%), 76 lymphomas (27%), 65 germ cell and gonadal tumors (26%), 37 epithelial tumors (13%), 14 soft tissue sarcomas (5%), 7 osteosarcomas (2%), 5 brain tumors (2%), and 16 other cancers (6%). The number of patients with the three most common tumor types of leukemia, lymphomas, and germ cell and gonadal tumors also generally increased each year (Figure 1B). The proportion of cancer types in patients aged 15–19 years was similar to the proportion of cancer types in all patients, while the proportions of patients with germ cell and gonadal tumors and epithelial tumors were lower in patients aged 0–14 years; meanwhile, the proportions of patients with soft tissue sarcoma, osteosarcoma, and brain tumors were noted to increase (Figure 2A).

Based on the regional distribution of patients, approximately half were from eastern China (49.8%), followed by western China (41.1%), and a small number from central China (9.1%). The numbers of patients in eastern and western China were similar. By analyzing the distribution of cancer types in patients undergoing fertility protection in these two regions, among the three main tumor types in western China, the proportion of patients with germ cell and gonadal tumors was the highest (28.3%), followed by lymphoma (24.8%) and leukemia (15.9%). Lymphoma (35.8%) accounted for the highest proportion of the three primary tumor types in eastern China, followed by leukemia (25.5%), and germ cell and gonadal tumors (17.5%). The number of patients with soft tissue sarcoma, osteosarcoma, and brain tumors was all higher in western China than that in eastern China (Figure 2B).

Population's sociocultural characteristics were analyzed and correlated with sperm cryopreservation. Patient age (*p* = 0.77), ethnicity (*p* = 0.52), and region (*p* = 0.38) were not relevant factors for sperm cryopreservation. We randomly surveyed 40 patients on fertility‐related issues and learned that they were all recommended by doctors to visit sperm banks for fertility preservation and that they were concerned about their fertility risks and hoped to have children in the future. If testicular tissue was not found to contain sperm, 47.5% of the patients wanted to retain the testicular tissue for potential development of technology in which spermatogonial stem cells (SSCs) could be used to induce sperm production in vitro (Table 1).

**TABLE 1:** Population characteristics, preservation of semen samples, and fertility‐related issues in patients.

	Sample stored, *n* (%)	Sample not stored, *n* (%)	*p*‐value
Age			
≤14 years	11 (84.6%)	2	0.77
15–19 years	229 (87.4%)	33	
Nationality			
Han	227 (87%)	34	0.52
Minority nationality	13 (92.9%)	1	
Region			
Eastern^a^	116 (84.7%)	21	0.38^a^
Central	24	1	
Western^a^	100 (88.5%)	13	
Fertility‐related issues (40 patients surveyed)	*n* (%)		
Doctor(s) recommended sperm banking			
Yes	40 (100%)		
No	0		
Worried about fertility risks			
Yes	40 (100%)		
No	0		
Goal to have a child			
Yes	40 (100%)		
No	0		
Willing to freeze testicular tissue for future use			
Yes	19 (47.5%)		
No	21 (52.5%)		

^a^ The chi‐squared test was applied for comparisons between ≤14 years and 15–19 years, Han and Minority nationality, eastern and western.

### 3.2 Semen parameters

Of the 275 patients, sperm was successfully frozen for 244 patients (88.7%). Five patients (1.8%) had immotile sperm in their semen; thus, their sperm was not frozen; 22 patients (8%) presented with azoospermia, and 4 patients (1.5%) were unable to provide a semen sample.

Tables 2, 3, 4 show semen parameters for the first ejaculate for the whole study population.

**TABLE 2:** Semen parameters by cancer type.

	LE	LY	GCGT	ET	STS	Other	All	Comparison (*p*‐value)
Number	55	76	65	37	14	16	263	
No. azoospermia	13	4	1	0	1	1	20	
No. no motility sperm	4	1	0	0	0	1	6	
No. unable to produce sample	0	1	1	1	0	1	4	
Age (years)	17.22 ± 1.5	17.32 ± 1.46	17.77 ± 1.27	17.51 ± 1.35	16.64 ± 1.91	17.75 ± 1.24	17.43 ± 1.44	NS
BMI	21.06 ± 4.41	21.6 ± 3.85	20.45 ± 2.72	19.87 ± 3.24	20.35 ± 2.63	19.94 ± 2.61	20.83 ± 3.57	NS
Semen volume (mL)	3.0 ± 1.95	2.99 ± 1.76	3.25 ± 1.59	2.45 ± 1.28	2.23 ± 1.36	3.09 ± 2.02	2.94 ± 1.71	NS
Sperm concentration (10^6^/mL)	19.33 ± 29.39	56.77 ± 57.55	33.91 ± 35.98	49.2 ± 39.83	48.15 ± 35.39	18.27 ± 13.29	40.1 ± 44.65	LE < LY, *p* < 0.001
								LE < ET, *p* = 0.004
								Other < LY, *p* < 0.001
Initial forward motility	21.65 ± 23.22	37.44 ± 18.84	40.48 ± 20.21	42.0 ± 16.12	41.23 ± 23.05	28.2 ± 17.99	35.56 ± 20.96	LE < LY, *p* = 0.001
								LE < GCGT, *p* < 0.001
								LE < ET, *p* < 0.001
								LE < STS, *p* = 0.03
Post‐thaw sperm concentration (10^6^/mL)	23.44 ± 24.57	39.41 ± 38.67	24.85 ± 24.18	31.26 ± 28.88	34.08 ± 23.47	13.95 ± 10.81	30.31 ± 30.58	LE < LY, *p* = 0.022
								GCGT < LY, *p* = 0.013
								Other < LY, *p* = 0.006
Post‐thaw forward motility	19.11 ± 13.22	23.11 ± 12.77	23.48 ± 13.24	17.82 ± 11.62	27.18 ± 16.94	18.31 ± 10.83	21.72 ± 13.02	NS
Recovery rate (%)	46.37 ± 23.83	51.55 ± 18.71	53.12 ± 18.86	42.23 ± 18.49	55.27 ± 20.13	56.26 ± 15.66	50.32 ± 19.47	NS
Number of cryotubes (mean)	5	4	5	4	3	3	4	

*Note*: Values are mean ± standard deviation (SD).

Abbreviations: ET, epithelial tumors; GCGT, germ cell and gonadal tumors; LE, leukemia; LY, lymphomas; NS, not significant, *p* > 0.05; STS, soft tissue sarcomas.

**TABLE 3:** Correlation of semen parameters with age.

	≤14 years old	15 years old	16 years old	17 years old	18 years old	19 years old	All	Comparison (*p*‐value)
Number	13	22	35	56	73	76	275	
No. azoospermia	3	4	6	4	4	1	22	
No. no motility sperm	0	0	0	1	1	3	5	
No. unable to produce sample	0	1	1	1	1	0	4	
BMI	19.38 ± 2.51	20.31 ± 3.41	20.13 ± 4.21	20.84 ± 3.71	21.25 ± 4.09	21.24 ± 2.62	20.87 ± 3.57	NS
Semen volume (mL)	1.92 ± 1.12	2.24 ± 1.19	2.68 ± 1.8	2.90 ± 1.66	2.97 ± 1.33	3.42 ± 2.03	2.93 ± 1.69	≤14 years old <19 years old, *p* = 0.01 15 years old <19 years old, *p* = 0.023
Sperm concentration (10^6^/mL)	29.52 ± 32.82	46.95 ± 66.0	49.21 ± 61.66	41.20 ± 37.75	37.36 ± 39.35	40.42 ± 38.57	40.90 ± 44.70	NS
Initial forward motility	31.54 ± 28.12	27.29 ± 21.6	29.68 ± 24.22	38.37 ± 21.33	38.09 ± 18.98	38.36 ± 18.87	35.82 ± 21.12	NS
Post‐thaw sperm concentration (10^6^/mL)	24.8 ± 21.77	39.49 ± 60.86	37.14 ± 34.15	32.15 ± 26.95	27.97 ± 26.16	28.71 ± 23.11	30.88 ± 30.56	NS
Post‐thaw forward motility	22.33 ± 17.22	17.0 ± 12.88	19.38 ± 15.14	26.58 ± 13.92	23.42 ± 11.20	19.76 ± 11.95	21.93 ± 13.12	NS
Recovery rate (%)	51.99 ± 22.34	42.57 ± 22.7	50.56 ± 26.91	54.39 ± 19.68	50.36 ± 17.83	48.06 ± 19.78	49.92 ± 20.56	NS

*Note*: Values are mean ± standard deviation (SD).

Abbreviation: NS, not significant, *p* > 0.05.

**TABLE 4:** Comparison of semen parameters before and after treatment.

	Before treatment	After treatment	Comparison (*p*‐value)
Number	93	49	
No. azoospermia	3	7	
No. no motility sperm	0	5	
No. unable to produce sample	0	1	
Semen volume (mL)	3.07 ± 1.83	2.65 ± 1.55	NS
Sperm concentration (10^6^/mL)	49.90 ± 52.80	22.15 ± 31.53	*p* < 0.001
Initial forward motility	40.01 ± 18.14	24.62 ± 20.60	*p* < 0.001
Post‐thaw sperm concentration (10^6^/mL)	32.67 ± 34.15	19.98 ± 21.45	NS
Post‐thaw forward motility	23.11 ± 12.05	14.96 ± 9.91	*p* = 0.001
Recovery rate (%)	50.79 ± 19.22	48.94 ± 20.69	NS

*Note*: Values are mean ± standard deviation (SD).

Abbreviation: NS, not significant, *p* > 0.05.

As shown in Table 2, semen parameters were analyzed in 263 patients with tumors, including leukemia, lymphomas, germ cell and gonadal tumors, epithelial tumors, soft tissue sarcomas, and other tumors. There were 20 cases of azoospermia; of these, 13 were diagnosed with leukemia and 4 were diagnosed with lymphoma. Six semen samples contained immotile sperm, and the highest proportion of immotile sperm was observed in patients with leukemia (4 cases). No significant differences were found in terms of age, semen volume, and recovery rate among different tumor types, with mean values of 17.43 ± 1.44 years, 2.94 ± 1.71 mL, and 50.32% ± 19.47%, respectively. The average number of cryotubes were four. Before freezing, sperm concentration and progressive motility varied by disease type. In a further multigroup analysis, sperm concentrations were significantly lower in patients with leukemia compared with those with lymphoma (*p* < 0.001) or epithelial tumors (*p* = 0.004). In contrast, the sperm concentrations in patients with leukemia were not significantly different from that with germ cell and gonadal tumors (*p* > 0.05). Lower semen concentrations in patients with germ cell and gonadal tumors were associated with unilateral orchiectomy in >70% patients prior to cryopreservation. Sperm progressive motility was significantly reduced in patients with leukemia compared with those with lymphoma (*p* = 0.001), germ cell and gonadal tumors (*p* < 0.001), epithelial tumors (*p* < 0.001), or soft tissue sarcomas (*p* = 0.03). After freezing, sperm progressive motility did not differ significantly among tumor types, whereas sperm concentrations varied by disease type. In a further multigroup analysis, patients with lymphomas were found to have significantly higher sperm concentrations compared with patients with leukemia (*p* = 0.022) and those with germ cell and gonadal tumors (*p* = 0.013).

As shown in Table 3, sperm concentration and sperm motility in 275 patients of different ages with tumors were not significantly different before and after freezing, and no significant difference was seen in the recovery rate. The average recovery rate was 49.92% ± 20.56%. Semen volume increased significantly with age (≤14 years old <19 years old, *p* = 0.01; 15 years old <19 years old, *p* = 0.023), which is consistent with previous report.^28^ This may also reflect a still incomplete postpubertal maturation of the accessory glands of the genital tract. The average semen volume was 2.93 ± 1.69 mL, and 71.6% of the semen volume was ≥2 mL.

Due to partial data loss, semen analysis was performed in 142 patients before and after treatment. Tumor treatment exerted a greater impact on sperm, and of the 10 cases of azoospermia, 7 (30.4%) were seen after treatment and 3 (2.5%) were observed before treatment. All cases of low motility were noted only after treatment (5 cases). Significant differences were found in sperm concentration (*p* < 0.001) and sperm progressive motility before and after treatment (*p* < 0.001). After thawing, significant differences were also seen in sperm progressive motility before and after treatment (*p* = 0.001). However, no significant differences were observed in sperm concentration and recovery rates in thawed sperm before and after treatment (Table 4).

## 4 DISCUSSION

Our study is the largest retrospective study of sperm cryopreservation in male adolescents with cancer (*n* = 275) in China. Successful sperm preservation was achieved in 244 patients (88.7%), demonstrating the feasibility of semen cryopreservation in male adolescents with cancer. In the United Kingdom, a study of 55 patients between the ages of 13 and 21 who were treated for potential gonadotoxicity and who visited a sperm bank for fertility preservation between 1997 and 2001 reported that 67% were able to cryopreserve their sperm.^29^ In the same United Kingdom study, 66% of 180 patients aged 13–17 years between 1995 and 2009 successfully cryopreserved their sperm.^30^ Another study in the United Kingdom reported 238 male adolescents with cancer aged <20 years from 1991 to 2000, of whom 205 (86.1%) underwent successful semen preservation.^31^ A study in the Netherlands reported successful semen cryopreservation in 66.25% of 80 boys aged 13.7–18.9 years from 1995 to 2005.^32^ In a French study of 156 patients (mean 17.81 ± 0.14 years of age) treated between January 1984 and December 2006, 88.5% were eligible for sperm cryopreservation.^33^ The largest retrospective study of fertility preservation in male adolescents with cancer, also in France, reported sperm cryopreservation in 3616 (83%) of 4345 patients aged 11–20 years between 1973 and 2007.^34^ In a Danish study of 25 boys aged 13–18 with cancer between January 01, 1995 and July 31, 1998, 21 boys (84%) provided semen samples for cryopreservation.^35^

In addition, in our study, 5 patients had immotile sperm; thus, their sperm was not frozen; 22 patients presented with azoospermia and 4 patients were unable to provide a semen sample. For masturbation patients, testicular sperm extraction (TESE), micro‐testicular sperm extraction (micro‐TESE), or orchiectomy (onco‐mTESE) are options for fertility preservation if there are immotile sperm or no sperm in the semen specimens.^36^, ^37^, ^38^ The urgency of disease treatment and the lack of medical technology may be the reasons why azoospermia patients do not undergo surgical sperm retrieval.

Among the global adolescent cancer types in individuals aged 15–19 years, lymphoma was the most common cancer in all regions at 22.5%, epithelial tumors were the second most common at 21.3%, and leukemia was the third most common at 15.4%; germ cell and gonadal tumors accounted for 12.0%, CNS tumors accounted for 10.7%, bone tumors accounted for 7.8%, and soft tissue sarcomas accounted for 7.0%.^39^ In this study, in patients aged 15–19 years, lymphomas accounted for 27.9%, germ cell and gonadal tumors accounted for 24%, leukemia accounted for 20.2%, epithelial tumors accounted for 13.7%, soft tissue sarcoma accounted for 4.6%, osteosarcoma accounted for 1.9%, and brain tumors accounted for 1.5%. The cancer type associated with the highest rate of sperm cryopreservation was lymphoma, and patients with lymphoma and leukemia underwent cryopreservation at a similar rate to the proportion of those cancer types. The proportion of fertility preservation in patients with germ cell and gonadal tumors was also noted to be higher, which is important considering the direct link of these cancers with reproductive ability. The proportion of fertility preservation in patients with epithelial tumors and CNS tumors was relatively low compared with the proportion of those cancer types. In our study, epithelial tumors mainly include nasopharyngeal carcinoma, thyroid cancer, liver cancer, lung cancer, and stomach cancer. Liver, lung, and stomach cancers have a high mortality rate and are poorly preserved. Due to low mortality and little effect of treatment on reproduction, the number of patients with high incidence of thyroid cancer chose sperm preservation is small. The incidences of bone tumors and soft tissue sarcomas were relatively low, but a certain proportion of patients still underwent fertility preservation. The number of patients with soft tissue sarcomas, bone sarcomas, and brain tumors are higher in western China than in eastern China. It may be that the Sichuan Human Sperm Bank in western China has actively promoted the male tumor fertility preservation over the past 5 years. This includes active communication with oncologists, scientific lectures for patients, and free semen and blood hormone testing during follow‐up, so the self‐efficacy of the sperm bank plays an active role. At the Sichuan Human Sperm Bank, the number of male tumor patients (0–19 years old) was 70, a rate of 61.9% in western China. The China Cancer Center indicated in the latest national cancer report in 2018 that the incidence of malignant tumors was highest in the eastern region followed by the central and western regions. Although the economic level and medical level of the eastern China are higher than that of the western China, the number of patients from the eastern and western regions was similar and accounted for 90.9% of the total, while the remaining patients were from central China. Therefore, the degree of development in the region may not be the main reason affecting fertility preservation, and self‐efficacy of sperm banks may increase the number of patients with fertility preservation and expand the types of tumors.

In terms of male adolescents with cancer, the proportions of Han and minorities who underwent fertility preservation were consistent with the ratios of Han and minority populations; thus, the inclusion of different ethnicities may not be the cause of low fertility preservation. In our study, 51% of the patients had a high school education, 24.2% had less than a high school education, 9.5% had post‐high school training/no bachelor's degree, and, finally, 15.3% of the patients had a college degree. The low age group that underwent fertility preservation comprised patients of only 0–14 years of age, and of these, 3 cases (4.7%) experienced nocturnal emission and were able to successfully provide semen samples. The youngest age was 13 years old. Whether fertility preservation should be required if the patient is young, has not produced semen, or has immature testicular tissue is yet to be established. For prepubertal male tumor patients, ASCO, JSCO, and the Chinese consensus on male fertility preservation suggest that there is no standard of fertility preservation regimen, and the only method of fertility preservation that can be considered is to cryopreserve human testicular tissue prior to treatment, which is still in the research stage.^40^, ^41^, ^42^, ^43^ Some studies have reported that an important milestone in immature testicular tissue (ITT) transplantation is the production of sperm from ITT autologous transplantation; additionally, the first nonhuman primate was born after intracytoplasmic sperm injection (ICSI).^44^ In Europe and the United States, more than 1033 young patients (age range 3 months to 18 years) have already undergone testicular tissue retrieval and storage for fertility preservation.^45^ Testicular tissue cryopreservation prior to hematopoietic stem cell transplantation in three cases of severe β‐thalassemia prepuberty boys was reported for the first time in 2022 in China.^46^ In our study, if the testicular tissue was devoid of sperm, approximately half of the patients were willing to retain their testicular tissue in case future technology could use SSCs to induce sperm production in vitro. This demonstrates that when patients realize that they are at risk of infertility, they will be concerned and more willing to undergo fertility protection and want to become parents, which shows the demand for future fertility. As the mortality rate of cancer patients is reduced and the survival period is prolonged, the improvement in the quality of life of patients after treatment and the needs for future reproduction are becoming the focus of attention. Research also found that physician recommendations play an important role, as patients reported that they were not aware of fertility protection prior to their physician recommendation. A multidisciplinary collaboration should be established between sperm banks and andrology and oncology departments, and implementation of fertility protection training programs should be implemented for doctors and nurses. Moreover, medical procedures between different hospitals and departments should also be established. To date, assisted reproductive technology services are not covered by the medical insurance system at the national level. Sperm cryopreservation as part of fertility treatment costs thousands of RMB, which is a heavy burden for low‐income people. Finally, financial incentives should be provided to direct patients to sperm banks for fertility assessment and sperm cryopreservation.

For fertility preservation, enough normal specimens should be stored for 10 or more inseminations, to ensure a good chance of pregnancy.^27^ Scholars in the United States have made the recommendation that for patients with cancer who want to achieve an eventual pregnancy in their spouses, they should store specimens for at least six intrauterine inseminations and also one or two for later IVF‐ICSI.^47^ In our study, the average number of cryotubes were four. Although there were variations in the frozen volume of semen specimens among the 16 human sperm banks, the number of cryotubes were far from adequate. Chinese experts suggest that the frozen volume of semen specimen per tube can be appropriately reduced and the number of cryotubes are increased to improve the success rate of later assisted reproductive technologies (ART).^48^

For the cryopreservation of sperm, 96% of the patients chose to continue freezing the specimens, and 4% of the patients choose to destroy the specimens, which is much lower than in adult patients with cancer.^49^ This is consistent with our findings. Thus far, the youngest surviving patient in our study was only 15 years of age, and the oldest was not older than 30 years. They may still be in the period of reproductive demand. Therefore, this further reflects the importance of fertility preservation in male adolescents with cancer, avoids the impact of later treatment on reproduction, and realizes the needs of future fertility. Due to lacking follow‐up data, the reasons for patient destruction of specimens, the use of specimens, and pregnancy outcomes are not known.

Our results were evaluated in the context of the strengths and limitations of our study design. The multi‐institutional nature of our cohort reduced confounding factor. No research on semen cryopreservation in children and adolescents with cancer in China has been published. In our study, no significant differences were observed in age or semen volume among patients with different tumor types, but significant differences were found in semen parameters before and after freezing. Leukemia patients had lower sperm concentration and progressive motility and also had lower concentrations after freezing, with the highest rate of azoospermia (65%) and the highest rate of low motility (66.7%). Possibly due to the urgency of treatment for leukemia and the lack of awareness of fertility preservation among patients, most of them received chemotherapy before sperm cryopreservation. Testicular cancers had the lowest total sperm output consistent with previous reports,^50^, ^51^ which is consistent with our findings. Germ cell and gonadal tumors showed low pre‐freeze and post‐thaw sperm concentrations, which may be related to the unilateral orchidectomy of tumor‐bearing testis prior to cryostorage. However, no significant difference was seen in the recovery rate among tumor types, which indicates that tumor type does not exert an impact on the freezing effect. Analysis of semen parameters before and after freezing at different ages showed that semen volume increased significantly with age, while semen concentration and progressive motility were not correlated with age. After freezing, concentrations, progressive motility, and recovery rates also did not differ significantly by age. A limitation of this study was the lack of a control group in which normal values could be determined in adolescents without malignant disease. In our study, approximately half of the patients abstained for more than 7 days due to their younger age. The mean BMI of the patients was 20.87 ± 3.57, 46.8% of the patients had a BMI <20 and 9.2% had a BMI >25. Studies have reported that both underweight and overweight were significantly associated with lower semen quality.^52^ In cases of gonadotoxic (chemotherapy and radiation) exposure of the testes with ongoing spermatogenesis (adolescent or adult), a significant reduction in sperm concentration within 2 months was observed.^53^ SSCs, and/or morphological damage or dysfunction of Sertoli cells, would result in permanent azoospermia. Therefore, it was not possible to assess whether semen quality was normal or whether it was affected by the cancer or the treatment. Data are accumulated in many different laboratories and over long periods of time, which can subject results to significant changes in laboratory techniques and data collection. There are differences in the sperm cryoprotectants used among 16 human sperm banks. However, each cryoprotectant has a freezing process that matches it for optimal freezing result. The recovery rate of donor specimens after thawing from 16 human sperm banks was essentially about 60%, which reduced the difference in freezing levels. Nonetheless, previous studies described baseline semen parameters in adolescents with malignancies, thereby helping to establish reference ranges for these patients.^30^, ^34^ This current study supports and extends the findings of these previous studies.

This study provides the most comprehensive national statistical census and review of fertility preservation in male adolescents with cancer trends, quantity, and cancer types. These data suggest that sperm production may occur even in very young adolescent boys. Although some providers or family members may overlook the potential for fertility preservation in these adolescent boys, current data underscore the importance of discussing and offering fertility preservation to even the youngest patients. We should also advocate for fertility preservation prior to gonadotoxic therapy or other procedures that may impair future fertility.

Fertility preservation for men with cancer started late in China and is relatively backward compared to that in developed countries.^34^, ^54^, ^55^, ^56^ In our study, although the number of patients who undergo fertility preservation has continued to increase yearly, only about 1% of male adolescents with cancer receive fertility preservation. Next, a large‐scale questionnaire survey will be conducted to achieve a comprehensive understanding of the factors affecting fertility preservation. Further research needs to establish a systematic follow‐up mechanism to strengthen the establishment of reproduction concerns in oncology patients, training of relevant medical personnel, and promotion of fertility preservation awareness in patients and their families, including detailed surgical methods, radiotherapy and chemotherapy regimens and doses, determination of semen quality at regular follow‐up (every year), semen recovery time for different tumor types, the use of frozen specimens, patient pregnancy outcomes, and offspring health. We should also improve our systematic understanding of how tumors affect fertility, comprehensively assess patient outcomes, and provide guidance on fertility protection to cancer patients with different conditions.

## AUTHOR CONTRIBUTIONS

**Shasha Liu:** Conceptualization (equal); formal analysis (equal); methodology (equal); writing – original draft (equal). **Qiling Wang:** Data curation (supporting); investigation (equal); resources (supporting). **Wenbing Zhu:** Conceptualization (supporting); data curation (supporting). **Zhou Zhang:** Conceptualization (supporting); data curation (supporting). **Wenhao Tang:** Conceptualization (supporting); data curation (supporting). **Huiqiang Sheng:** Conceptualization (supporting); data curation (supporting). **Jigao Yang:** Conceptualization (supporting); data curation (supporting). **Yushan Li:** Conceptualization (supporting); data curation (supporting). **Xiaowei Liang:** Conceptualization (supporting); data curation (supporting). **Tianqing Meng:** Conceptualization (supporting); data curation (supporting). **Zhiqiang Wang:** Conceptualization (supporting); data curation (supporting). **Faxi Lin:** Conceptualization (supporting); data curation (supporting). **Hao Dong:** Conceptualization (supporting); data curation (supporting). **Xiaojin He:** Conceptualization (supporting); data curation (supporting). **Xianglong Jiang:** Conceptualization (supporting); data curation (supporting). **Shanjun Dai:** Conceptualization (supporting); data curation (supporting). **Aiping Zhang:** Conceptualization (supporting); data curation (supporting). **Chunying Song:** Conceptualization (supporting); data curation (supporting). **Zuowen Liang:** Conceptualization (supporting); data curation (supporting). **Feng Zhang:** Conceptualization (supporting); data curation (supporting). **Xiaojun Wang:** Conceptualization (supporting); data curation (supporting). **Peiyu Liang:** Conceptualization (supporting); data curation (supporting). **Guihua Gong:** Conceptualization (supporting); data curation (supporting). **Xiaohong Huai:** Conceptualization (supporting); data curation (supporting). **Yanyun Wang:** Conceptualization (supporting); data curation (supporting). **Fuping Li:** Funding acquisition (equal); project administration (lead); resources (supporting); supervision (equal); validation (equal); writing – review and editing (lead). **Xinzong Zhang:** Investigation (lead); project administration (lead); resources (lead); supervision (equal); validation (equal); writing – review and editing (lead).

## FUNDING INFORMATION

This work was supported by Sichuan Science and Technology Program (2022YFS0045).

## CONFLICT OF INTEREST STATEMENT

The authors have no conflict of interest to declare.

## Data Availability

The data that support the findings of this study are available from the corresponding author upon reasonable request.
